# Pretreatment with levosimendan improves outcome in off-pump coronary artery bypass grafting

**DOI:** 10.1186/1749-8090-8-S1-O159

**Published:** 2013-09-11

**Authors:** I Husedzinovic, N Bradic

**Affiliations:** 1 Clinic of Anesthesiology, Reanimatology and Intensive Medicine, University Hospital Dubrava, Zagreb, Croatia

## Objective

Levosimendan, calcium sensitizer, induces contractility by binding to troponin C without increasing intracellular calcium concentration. We tested the hypothesis that levosimendan could produce early and prolonged beneficial effect on left ventricular (LV) systolic function in patients with normal LV function undergoing off-pump coronary artery bypass grafting (OPCABG).

## Methods

Loading dose of levosimendan at low-dose (12 mcg/kg), high-dose (24 mcg/kg) or placebo were administered in thirty-one patients in randomized controlled study. Bolus thermodilution by pulmonary artery catheter used for cardiac output (CO) measuring. Numerical data were described by median. To compare the three independent groups (low or high-dose levosimendan or placebo) the Kruskal-Walis test was applied. For comparing time points in one group, we tested the differences in time within each group with the Friedman test.

## Results

Significant increase in CO occurred after low-dose (p = 0.001) and high-dose levosimendan (p < 0.001). CO was higher in all measurements in patients receiving low-dose levosimendan versus patients received placebo, but didn't rich significance. In patients receiving low-dose levosimendan, compared with baseline measurement, CO was higher 20 minutes after infusion (p = 0.080) and 48 h after surgery (p=0.067). Similar alteration in CO measurements occurred after high-dose comparing with low-dose levosimendan. Furthermore, CO was significantly higher in patients receiving high-dose levosimendan compared with those receiving placebo, 20 min after infusion (p = 0.021) and 48 h after surgery (p = 0.006). Compared with baseline measurement, all values of CO were significantly higher in patients treated with high-dose levosimendan (p = 0.005 for all).

## Conclusion

Levosimendan produces early and prolonged beneficial effect on left ventricular performance in patients with preoperative normal LV function undergoing OPCABG surgery, and this effect is dose dependent.

